# Near-infrared fluorescent nanoprobe enables noninvasive, longitudinal monitoring of graft outcome in RPE transplantation

**DOI:** 10.3389/fmed.2025.1583790

**Published:** 2025-05-09

**Authors:** Guanzhou Di, Chen Lu, Mengting Xue, Limin Zheng, Weiqi Li, Runmin Xie, Xinpei Yuan, Xu Zhen, Min Wu, Xiying Mao, Songtao Yuan

**Affiliations:** ^1^Department of Ophthalmology, The First Affiliated Hospital of Nanjing Medical University, Nanjing Medical University, Nanjing, Jiangsu, China; ^2^Department of Ophthalmology, The First Affiliated Hospital of Baotou Medical College, Inner Mongolia University of Science and Technology, Baotou, Inner Mongolia Autonomous Region, Nanjing, Jiangsu, China; ^3^MOE Key Laboratory of High Performance Polymer Materials & Technology and State Key Laboratory of Analytical Chemistry for Life Science, School of Chemistry & Chemical Engineering, Nanjing University, Nanjing, Jiangsu, China

**Keywords:** near-infrared fluorescent nanoprobe, RPE transplantation, *in vivo* tracking, immune rejection, macrophage

## Abstract

**Objectives:**

Retinal pigment epithelium (RPE) cell transplantation holds therapeutic promise for retinal degenerative diseases, but longitudinal monitoring of graft survival and efficacy remains clinically challenging. The aim of this study is to develop a simple and effective method for the therapeutic quantification of RPE cell transplantation and immune rejection *in vivo*.

**Methods:**

A nanoprobe was developed and modified to label donor RPE cells, and used to monitor the position and intensity of the fluorescence signal *in vivo*. Immunofluorescence staining and single-cell RNA sequencing (scRNA-seq) were used to characterize the cell types showing the fluorescence signal of the nanoprobe and to determine the composition of the immune microenvironment associated with subretinal transplantation.

**Results:**

The spatial distribution of the fluorescence signal of the nanoprobe corresponded with the site of transplantation, but the signal intensity decreased over time, while the signal distribution extended to the choroid. Additionally, the nanoprobe fluorescence signal was detected in the liver and spleen during long-term monitoring. Conversely, in mice administered the immunosuppressive drug cyclosporine A, the decrease in signal intensity was slower and the expansion of the signal distribution was less pronounced. Immunofluorescence analysis revealed a significant temporal increase in the proportion of macrophages with nanoprobe-labeled cells following transplantation. The stability and cell-penetrating ability of the nanoprobe enables the labeling of immune cell niches in RPE transplantation. Additionally, scRNA-seq analysis of nanoprobe-labeled cells identified MDK and ANXA1 signaling pathway in donor RPE cells as initiators of the immune rejection cascade, which were further amplified by macrophage-mediated pro-inflammatory signaling.

**Conclusion:**

Near-infrared fluorescent nanoprobes represent a reliable method for *in vivo* tracing of donor RPE cells and long-term observation of nanoprobe distribution can be used to evaluate the degree of immune rejection. Molecular analysis of nanoprobe-labeled cells facilitates the characterization of the dynamic immune cell rejection niche and the landscape of donor-host interactions in RPE transplantation.

## 1 Introduction

Retinal pigment epithelium (RPE) cell transplantation represents an important area of research for the treatment of retinal degenerative diseases and has shown efficacy in various clinical trials (1–3). However, the chronic loss of transplanted RPE cells remains a contentious and unavoidable issue in long-term follow up studies. This issue arises primarily due to two factors, namely the lack of clinical methods for tracing and quantifying transplanted cells, and the insufficient clinical evaluation of local immune rejection, due partly to the presence of the blood-retinal barrier (4, 5).

Currently, the most commonly used *in vivo* labeling technique for transplanted cells involves the use of reporter-gene systems (6, 7), which is suitable for short-term cell tracking, but not long-term. In addition, reporter-gene based imaging is limited to viable cells and cannot track the turnover of cells and cellular debris. As a novel class of biocompatible fluorescent nanoparticles used in cell tracking (8), semiconducting polymer (SP) nanoparticles show better photostability than reporter proteins and lower *in vivo* toxicity than inorganic quantum dots (9, 10). However, ratiometric imaging probes based on SP nanoparticles are not used for *in vivo* cell labeling and tracking as they require ultraviolet (UV) light excitation and emit in the visible range. Recently, a group modified SP-based nanoprobes, such as those based on poly[2,7-(9,9-dioctylfluorene)-alt-4,7-bis(thiophen-2-yl)benzo-2,1,3-thiadiazole] (PFO-DBT) nanoparticles, which can be excited with red light and exhibit dual SP near-infrared (NIR) emissions, were used to efficiently label the hypoxic regions of tumor tissues (11), establishing a precedent for the application of SP-based nanoprobes for *in vivo* labeling.

In our study, we enhanced the PFO-DBT nanoprobe by conjugating it with the TAT peptide (from the human immunodeficiency virus-1 (HIV-1) transactivator of transcription (TAT) protein) to facilitate cellular penetration (12–14). In the retina of mice transplanted with RPE cells, nanoprobe-labeled donor RPE cells were visualized by both NIR ratiometric fluorescence imaging and fundus fluorescence imaging. Additionally, due to its intracellular stability, the nanoprobe is capable of tracking the fate of transplanted cells and labeling the cells with which it interacts. Leveraging this capability, we performed single-cell transcriptome sequencing of nanoprobe-positive cells to characterize the dynamic immune cell rejection niche and the landscape of cell–cell interactions.

## 2 Materials and methods

### 2.1 Animals

Four-week-old, female BALB/c mice (strain ID: 221; Charles River Laboratories International Inc., Wilmington, MA, United States), were raised in a specific pathogen-free facility with a 12 h light/dark cycle at 28.5°C at Nanjing Medical University (Nanjing, China). Before all experimental procedures (surgical procedures and examinations), mice were anesthetized intraperitoneally with ketamine (80 mg/kg) and xylazine (4 mg/kg) and pupils were dilated with 1% cyclopentolate-HCl and 2.5% phenylephrine.

All animal experiments in this study conformed to the guidelines of the Care and Use of Laboratory Animals (published by the NIH publication No. 86–23, revised 1996), and were approved and consistently reviewed by the ethical review board of Nanjing Medical University (Approval No.: IACUC-2310105).

### 2.2 Synthesis and usage of the nanoprobe

PFO-DBT (0.5 g; MilliporeSigma, Burlington, MA, United States) and polyethylene glycol (PEG)-3000-maleimide (MAL) (1 mg; Ponsure Biotechnology Co., Ltd., Shanghai, China) were dissolved in 1 mL of tetrahydrofuran (THF), and then rapidly added to 10 mL of distilled water under sonication in a BKE-1008HT ultrasonic cleaner (Jinan Bakr Ultrasonic Technology Co., Ltd., Jinan, China). After further sonication for 5 min, the solution was stirred with a C-MAGHS7 magnetic stirrer (Guangzhou IKA Works, Guangzhou, China) at 700 rpm for 12 h to remove the THF. The human immunodeficiency virus-1 (HIV-1) transactivator of transcription (TAT) protein peptide (HIV-1 TAT peptide, RKKRRQRRRC, 1 mg) was dissolved in 100 μL dimethyl sulfoxide (DMSO) and stirred again for 8 h to couple the TAT peptide to PFO-DBT nanoparticles. Ultimately, DMSO was removed by ultrafiltration and the fluorescent nanoprobe was obtained.

### 2.3 Cell culture

The mouse microglial BV2 and human RPE ARPE19 cell lines were both obtained from American Type Culture Collection (ATCC, Manassas, VA, United States), and fetal RPE (fRPE) cells were isolated in our laboratory using an isolation procedure that fRPE was reviewed by the Ethics Committee of the First Affiliated Hospital of Nanjing Medical University (Approval No.:2017-SR-253.A2) and the human tissue experiments complied with the guidelines of the ARVO Best Practices for Using Human Eye Tissue in Research (November 2021). All cells were cultured in a 95% humidified incubator with 5% CO_2_, at 37°C. The complete growth medium for both BV2 and ARPE19 cells was Dulbecco’s modified Eagle’s medium (DMEM)-F12 (Gibco/Thermo Fisher Scientific Inc., Waltham, MA, United States) containing 10% fetal bovine serum (FBS; Invitrogen, Carlsbad, CA, USA) and 1% penicillin/streptomycin (100 U/mL; Invitrogen). The fRPE cells were maintained in DMEM-F12 medium (Gibco/Thermo Fisher Scientific Inc.) supplemented with 10% FBS (Invitrogen), SB-431542 (10 mM; MedChemExpress LLC, Monmouth Junction, NJ, United States), 1% N1 supplement (MilliporeSigma), penicillin/streptomycin (100 U/mL; Invitrogen), 1% non-essential amino acids (Invitrogen), Taurine (250 mg/mL; MilliporeSigma), hydrocortisone (20 g/mL; MilliporeSigma), and triiodothyronine (13 ng/L; MilliporeSigma).

### 2.4 Dissociation and expansion of fetal RPE

Fetal eyes were acquired from abortion donors in the First Affiliated Hospital with Nanjing Medical University, Jiangsu Women and Children Health Hospital. fRPE layers were mechanically separated from the choroid and cultured in The complete growth medium at 37°C in 5% CO_2_, and the RPE medium was changed every 2–3 days.These cells were termed Passage 0. Cultures were monitored daily under phase-contrast microscopy. Upon reaching 90–100% confluence, adherent cells were detached using 0.25% trypsin–EDTA and subsequently subcultured at a 1:3 split ratio in fresh complete medium. Cells at 90–100% confluence were harvested by trypsinization (0.25% Trypsin–EDTA), centrifuged at 300 × g for 5 min, and resuspended in freezing medium (90% FBS + 10% DMSO) at a density of 1 × 106 cells/mL. Cell suspensions were transferred to cryovials, gradually cooled using a freezing container, and ultimately stored in liquid nitrogen for long-term preservation.

### 2.5 Cell preparation before transplantation

To track the donor cells, we transduced the fRPE cells with lentiviral particles containing the GFP expression vector (Shanghai Genechem Co., Ltd., Shanghai, China) by adding it into the culture medium according to the manufacturer’s instructions. The cells with or without GFP were cultured in culture medium with a nanoprobe concentration of 20 μg/mL for 48 h, and the cells showed positive nanoprobe signal after washing 3 times with phosphate-buffered saline (PBS) solution.

### 2.6 Cell transplantation

Anesthetized animals were placed under a surgical microscope, and their pupils were pharmacologically dilated with 1% cyclopentolate-HCl and 2.5% phenylephrine. A small hole was made at the border between the sclera and cornea with a sterile 30-gauge½ needle to reduce intraocular pressure. A 32-gauge blunt-end microliter syringe (Hamilton, Reno, NV, United States) connected to a glass pipette containing 1 μL of cell suspension (10^5^ cells) was inserted into the sub-retinal space for graft delivery. The contralateral eye received the same treatment. Immediately following injection, we confirmed the success of each surgery through observation of retinal detachment using a surgical microscope. Only animals with successful sub-retinal injections were included in this study.

### 2.7 Cell counting kit-8 assay

The fRPE cells were seeded in 96-well plates at a density of 5,000 cells/well. The cell counting kit-8 (CCK-8; Beyotime, Shanghai, China) was used to determine cell viability following the manufacturer’s instructions.

### 2.8 The *in vivo* imaging system series Lumina III platform

The mice were divided into three groups: the blank group, the group with cyclosporine A (w/ CsA) and the group without cyclosporine A (w/o CsA). The mice in the w/CsA group were intraperitoneally injected cyclosporine A (5 mg/kg, MedChemExpress LLC) everyday; the mice in the w/o CsA group and blank group were intraperitoneally injected with corn oil (MedChemExpress LLC) at the same time. The anesthetized mice were placed on the imaging platform of the *In Vivo* Imaging System (IVIS) Series Lumina III system (PerkinElmer, Waltham, MA, United States), and the fluorescence signal of the mice was imaged with green light excitation at 520 and 680 nm absorption light. In addition, the intensity of fluorescence in the same area around the eyes of each mouse was recorded.

### 2.9 Small animal ophthalmic multimodal imaging system

The cornea of an anesthetized mice was pointed at the camera of the small animal ophthalmic multimodal imaging system. When observing the GFP fluorescence signal, it was excited with 488 nm light, and when receiving the fluorescence, it was blocked with a filter, allowing the light of 510–520 nm to be captured. Additionally, the nanoprobe fluorescence signal was imaged with green light excitation at 520 nm and blocked with a filter when receiving fluorescence, which allowed light at 670–690 nm to be captured. The fundus fluorescence images were collected at the same location in two channels. At the same time, the system was also used to perform optical coherence tomography (OCT) examination on the site of cell transplantation in the sub-retinal place of mice.

### 2.10 Confocal intravital microscopy

The distribution of probe signal in liver and spleen was visualized using the IVM-CMS3 intravital two-photon microscopy platform (IVIM Technology Inc., Daejeon, Korea). An anti-mouse CD31 antibody (IVIM Technology Inc.) was injected int. the mice via the tail vein to enhance image contrast. The mice were anesthetized after the angiography agent diffused, then the liver and spleen were surgically exposed and imaged using a two-photon microscope equipped with a water objective lens (20×). Imaging was performed in the Cy5 (663–733 nm, for probe) and GFP (503–626 nm, for CD31 antibody) channels.

### 2.11 Immunofluorescence microscopy

The fRPE cells were cultured on the coverslips with the nanoprobe for 48 h. At Day 1 (D1) and D14 after replenishing with fresh medium, the cells were washed 3 times with PBS before fixation in 4% paraformaldehyde at room temperature for 30 min. After another wash, 4′,6-diamidino-2-phenylindole (DAPI)-Fluoromount-G™ (Southern Biotech, Birmingham, AL, United States) was added to preserve the immunofluorescent labels. The eyeballs of the mice were fixed in FAS eyeball fixative solution (Servicebio, Beijing, China) for 1 h, washed three times with PBS, gradient-dehydrated with a sucrose solution, embedded in optimal cutting temperature compound (Sakura Finetek, Torrance, CA, United States), frozen in liquid nitrogen, and sectioned parallel to the direction of the optic nerve at 8 mm using a Leica CM1900 cryostat (Leica Microsystems GmbH, Wetzlar, Germany), to obtain retinal frozen sections of the location of the transplanted cells. Each slice was dried at room temperature for 1 h before staining, treated with PBST (PBS with 0.3% Triton-X 100) at room temperature for 1 h, and then blocked with 5% BSA for 1 h at room temperature before incubation with the appropriate primary antibody 12 h at 4°C. After a 5-min rinse with PBS, the slices were incubated with the appropriate secondary antibody for 1 h at room temperature. Cell nuclei were counterstained with DAPI-Fluoromount-G (Southern Biotech). Images were captured with a Leica DMi8 inverted microscope (Leica Microsystems GmbH) equipped with the Leica THUNDER imaging system (Leica Microsystems GmbH).

### 2.12 Flow cytometry

BV2 cell suspension was prepared and incubated with 5 μM of carboxyfluorescein diacetate succinimidyl ester (CFDA-SE; MedChemExpress LLC) in a total volume of 1 mL at room temperature for 30 min. After washing 3 times with PBS, CFDA-SE^+^ BV2 cells were co-cultured with ARPE19 cells with fluorescent nanoprobe directly in the same dish. In addition, fRPE cells and ARPE19 cells or BV2 cells were co-cultured using transwell inserts, in which fRPE cells were plated in the lower chamber followed by incubation with the probe after reaching confluence, and ARPE19 cells or CFDA-SE^+^ BV2 cells were plated in the upper chamber. The fRPE cells with positive probe signal in the lower chamber would not come into contact with cells in the upper chamber through the 0.4 μm pore of the insert membrane. After 12 h, cells in both chambers were detached by treatment with 0.25% trypsin–EDTA solution (Gibco/Thermo Fisher Scientific Inc.) and collected. After centrifugation at 300 × g for 5 min, cells were resuspended in 200 μL PBS with 2% FBS in preparation for flow cytometry analysis.

### 2.13 Bulk RNA-seq

Total RNA was extracted from fRPE cells and probe-labeled fRPE cells using TRIzol reagent (Invitrogen). Concentration and purity of the extracted RNA were detected using a Nano-Drop ND-1000 spectrophotometer (Nano-Drop Technologies, Wilmington, DE, United States). RNA sequencing libraries were constructed from 1 μg of total RNA using a modified TruSeq RNA Sample preparation kit (Illumina Inc., San Diego, CA, United States) protocol. Pass-filtered reads were mapped using STAR v2.6.1c and aligned to the human reference genome GRCh38.92 (15). The count table of the gene features was obtained using FeatureCounts (16). Normalization, differential expression analysis and TPM (transcript per million) values were calculated using EdgeR (16). Protein coding mRNAs with greater than 25 TPMs in two groups and a false discovery rate < 0.01 were selected.

### 2.14 Sample dissociation for single-cell RNA sequencing

After cell transplantation, mice were sacrificed by deep anesthesia at 1D and 5D post-surgery, respectively. Eyes were enucleated immediately and dissected in PBS with 2% FBS after euthanasia. For single-cell sequencing, bilateral eyes from 15 mice (*n* = 30 eyes) were pooled per time point. The anterior segments were removed, and posterior globe including sclera/choroid-RPE complex and neuroretina were preserved. The samples underwent mechanical and enzymatic digestion (2 × 10 min at 37°C) in a tissue dissociation solution (Papain Worthington Biochem Corp., Freehold, NJ, United States); L-cysteine (100 mg/15 mL), NaHCO_3_ (100 mg/mL) and DNase I (750 U/mg; BioFroxx GmbH, Einhausen, Germany), in DMEM medium [Basal medium, 4.5 g/L D-Glucose, HEPES, L-glutamine, phenol red (−), sodium pyruvate (−)]. Afterwards, cells were filtered, resuspended in PBS with 2% FBS. Probe^+^ cells were sorted using a BD FACSAria™ II SORP Flow Cytometer (BD Biosciences, San Jose, CA, United States). Sorted cells were collected in DMEM medium [Basal medium, 4.5 g/L D-Glucose, HEPES, L- glutamine, phenol red (−), sodium pyruvate (−)] supplemented with 20% FBS (Gibco/Thermo Fisher Scientific Inc.), ultimately adjusting the concentration to 1,000 cells/μL, for further processing in the 10 × genomics platform.

### 2.15 Single-cell RNA sequencing data analyses

We performed droplet-based single-cell encapsulation using the Chromium Next GEM Single Cell 3′ Reagent Dual Index Kit v3.1 (10x Genomics (Shanghai) Co., Ltd., Shanghai, China), followed by bar-coded reverse transcription to generate the bar-coded complementary DNA (cDNA) library. Two cDNA libraries were sent for high-throughput DNA Sequencing at Nanjing Jiangbei New Area Biopharmaceutical Public Service Platform Co., Ltd. (Nanjing, China), where the cDNA libraries were amplified and ligated with Illumina adaptor sequences, adjusted to appropriate concentration, pooled, and subjected to high-throughput DNA sequencing on the Illumina NovaSeq 6000 Platform (Illumina Inc.). The sequencing library format chosen was 150-bp pair-end. Software from Illumina was used to output the sequencing data in FASTQ format. Cell ranger 7.1.0 was used to map the sequencing reads in the FASTQ files onto a custom reference genome we prepared. The custom reference genome contains both the human (GRCh38) and the mouse (10 mm) reference genome sequences concatenated together. After processing, we generated a matrix with each row representing one human or mouse gene and each column representing one cell. The counts are integers representing the number of reads for the corresponding gene in that corresponding cell. The matrices were imported into Seurat 5.1.0 for further single-cell RNA sequencing (scRNA-seq) bioinformatics analysis and data visualization. For filtering cells, each cell had to satisfy all of the following criteria: (1) Minimum number of cells expressing a single gene needs to be more than 3; (2) the percentage of reads that mapped to mitochondrial DNA shall be less than 25%; and (3) the total number of unique genes with reads > 200 needs to be more than 500 genes. Proportion of cells belonging to human was analyzed and those with more than 95% were grouped into transplanted cells. The count matrix of all filtered 19,378 cells was normalized and subjected to dimension reduction by principal components analysis (PCA) performed on the normalized data. The principal components were used for subsequent analyses, including Uniform Manifold Approximation and Projection (UMAP) visualization and unsupervised clustering analyses. Seurat analysis was performed in the R software using Seurat 5.1.0. Differentially expressed genes (DEGs) were identified using the FindMarkers function implemented in the R package in Seurat. Cellchat was used to infer the cell–cell communication networks from single-cell transcriptome data (17).

The scRNA-seq data reported in this study have been deposited in the Genome Sequence Archive (Genomics, Proteomics & Bioinformatics 2021) in National Genomics Data Center (Nucleic Acids Res 2022) (18, 19), China National Center for Bioinformation/Beijing Institute of Genomics, Chinese Academy of Sciences (GSA: CRA019724) that are publicly accessible at https://ngdc.cncb.ac.cn/gsa.

### 2.16 Statistical analysis

All data shown represent at least three independent experiments. All experimental data are expressed as the mean ± standard error of mean (SEM). Statistical analyses were performed using the GraphPad Pro Prism 8.0 software (GraphPad Software Inc., San Diego, CA, United States). Statistical significance of the difference between sets of data was determined by unpaired two-tailed Student t-test followed by Bonferroni’s multiple comparisons test. *p* < 0.05 was considered statistically significant.

## 3 Results

### 3.1 Design and photophysical properties of the nanoprobe

**Figure 1:**
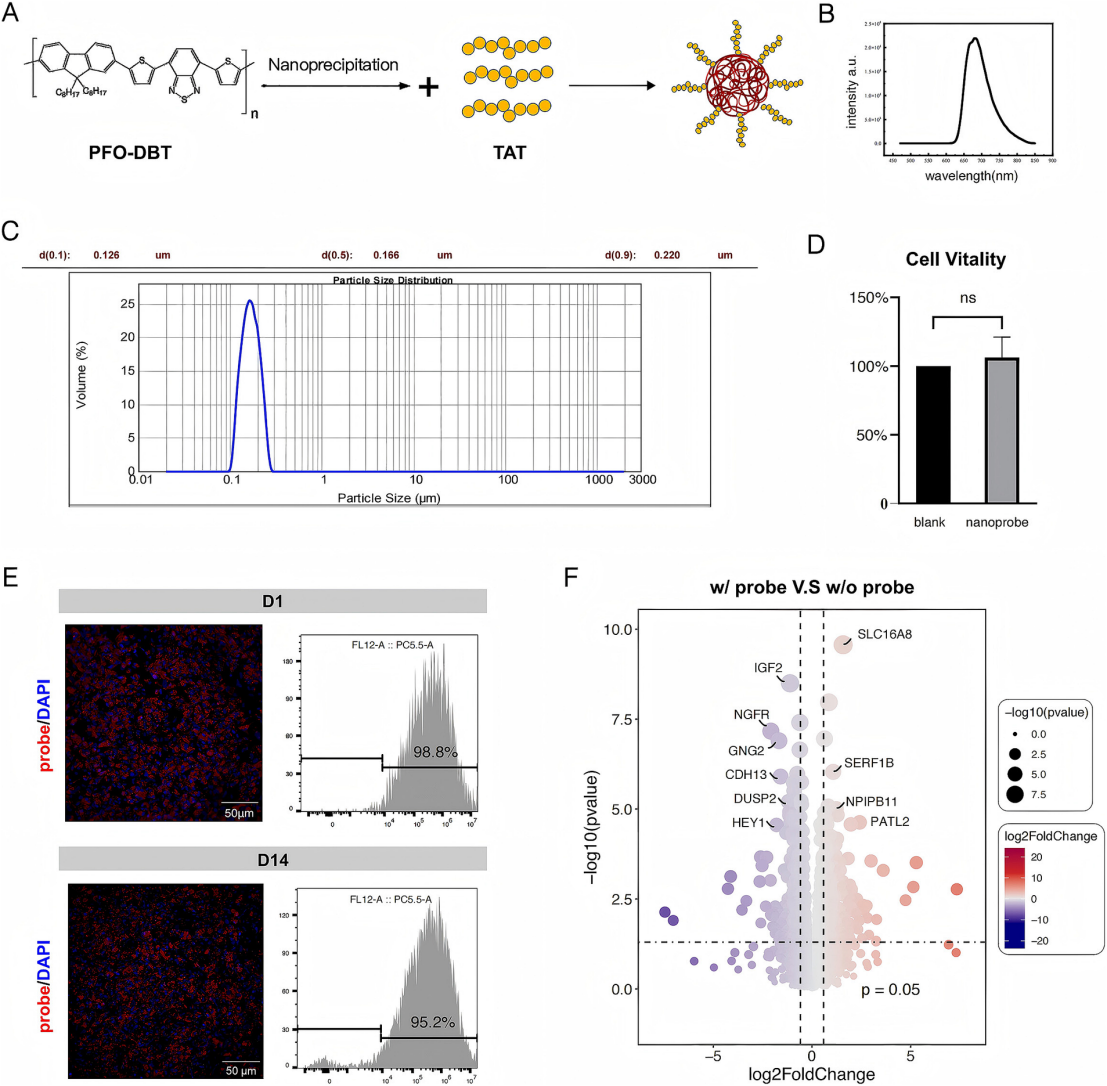
Properties and toxicity of the nanoprobe. **(A)** Schematic illustration of the nanoprobe preparation process. **(B)** Emission spectrum of the nanoprobe. **(C)** Particle size distribution of nanoprobes. **(D)** Comparison of cell viability between w/ probe group (nanoprobe) and w/o probe group (blank). **(E)** Immunofluorescence images of fRPE cells *in vitro* incubated with the nanoprobe and the proportion of probe-positive fRPE cells at D1 and D14 assessed by flow cytometry analysis. **(F)** Bulk RNA sequencing analysis of probe-labeled fRPE cells revealed gene expression profiles comparable to those of unlabeled cells.

The nanoprobe was synthesized by the nanoprecipitation method using the fluorescent SP PFO-DBT nanoparticles. Subsequently, the membrane-penetrating HIV-1 TAT peptide was conjugated to the nanoprobe (Figure 1A). Transmission electron microscopy analysis revealed that the synthesized nanoprobe had a diameter of 200 nm. Upon excitation at 630 nm, the nanoprobe showed maximal emission at 685 nm from the SP nanoparticles (Figures 1B,C).

In a toxicity assay, the viability of the cells incubated with the nanoprobe was comparable to that of the control group (Figure 1D). Effective labeling of the fRPE cells was achieved by incubation with the nanoprobe for 48 h, and the labeled cells remained stable for over 2 weeks *in vitro* (Figure 1E). The labeled cells were seeded and probe-labeled simultaneously, then plated into separate culture dishes. At designated time points, cells from randomly selected wells were fixed, imaged, and subjected to flow cytometry analysis. In addition, The bulk RNA sequencing of probe-labeled fRPE cells revealed no significant differences in genes related to RPE function and cell proliferation compared to unlabeled cells (Figure 1F; Supplementary Table S1; Supplementary Figure S1). These findings suggest the feasibility and safety of transplanting nanoprobe-labeled RPE cells.

### 3.2 Multimodal imaging of nanoprobe-labeled cells

**Figure 2:**
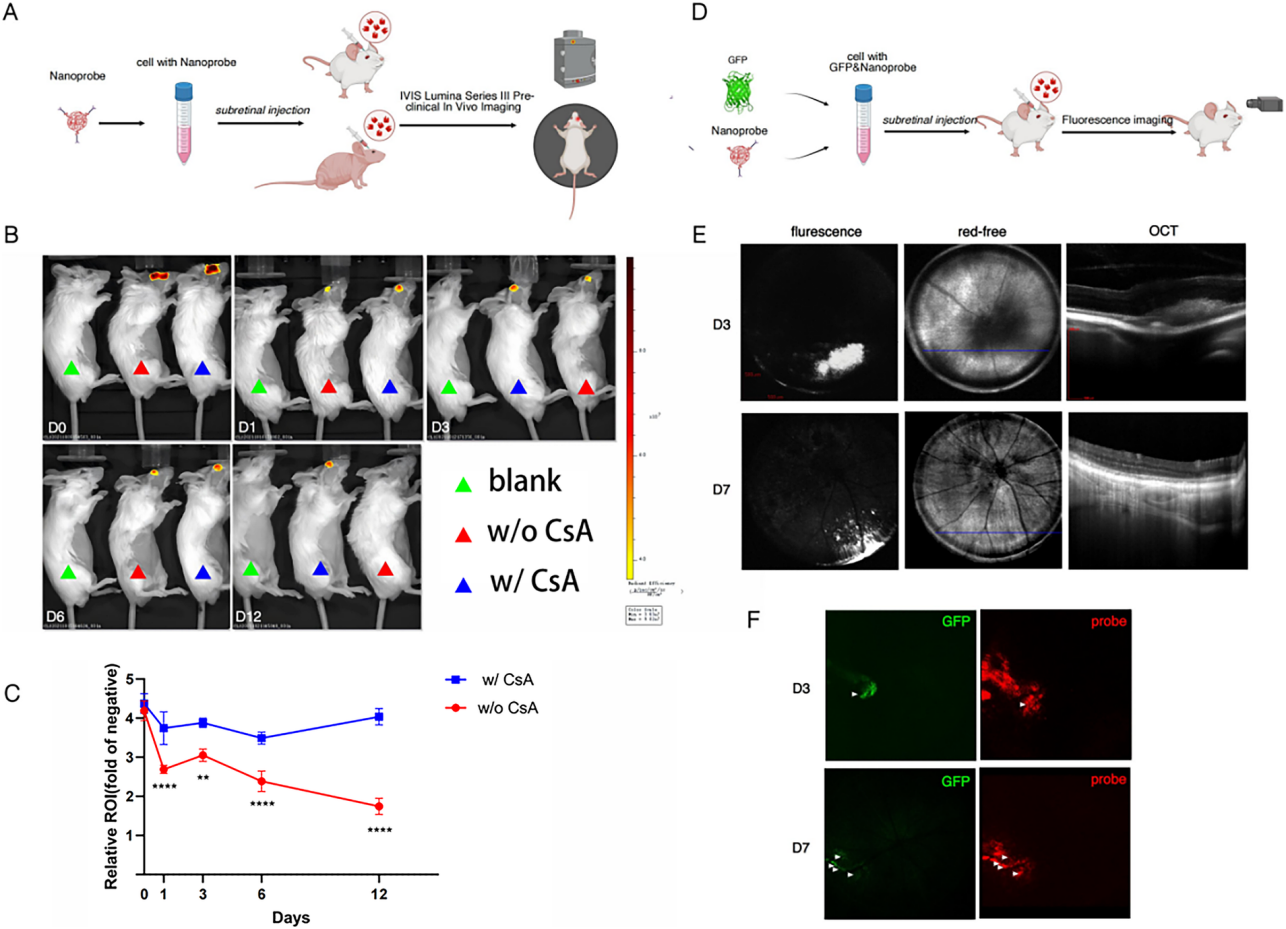
NIR ratiometric imaging and fundus multimodal imaging of nanoprobe-labeled cells. **(A)** Experimental scheme for nanoprobe imaging *in vivo* using the IVIS Series Lumina III imaging system (*n* = 3). **(B)** Representative images of mice in the blank, w/o cyclosporin A (CsA) and w/CsA groups at D0, D1, D3, D6, and D12 after cell transplantation captured by the IVIS Series Lumina III imaging system. **(C)** Line chart showing a slower decline of signal intensity in the w/CsA group compared to the w/o CsA group. **(D)** Experimental scheme for nanoprobe imaging by the small animal ophthalmic multimodal imaging system (*n* = 3). **(E)** Fundus fluorescence images and optical coherence tomography (OCT) of mice injected subretinally with nanoprobe-labeled cells at D3 and D7 after cell transplantation. **(F)** Fundus fluorescence images of both GFP and near-infrared (NIR) channels at different time points. The symbols **denote *p* = 0.01 and ****denote *p* = 0.0001.

We transplanted nanoprobe-labeled cells into the subretinal space of mouse eyes (Figure 2A). *In vivo* ratiometric imaging was performed at various timepoints after transplant (Figure 2B). The imaging results indicated that the nanoprobe signal was located in the center of the eye and showed a decreasing trend over time. Notably, the rate of signal decrease in immunosuppressive mice was slower than that observed in wild-type mice (Figure 2C). These findings demonstrate the effectiveness of the fluorescence-labeled nanoparticles produced by this nanoprobe labeling method for tracking transplanted cells.

However, this imaging modality does not allow fine localization of the region where the grafted cells are situated in the fundus. Subsequently, we used fundus multimodal imaging to track the transplanted cells (Figure 2D). Fundus fluorescence imaging revealed a substantial accumulation of fluorescence signal at the graft site, which corresponded to a highly reflective signal in the subretinal space observed in OCT images (Figure 2E). By D7 post-transplantation, the fluorescence signal at the graft site decreased, and the corresponding OCT imaging findings indicated that the grafted cells tended to distribute in a dispersed manner.

We conducted a comparative analysis of the imaging of GFP-transfected transplanted cells and found that the area and intensity of the GFP signal were significantly lower than those of the nanoprobe signal (Figure 2F). Two possible explanations for this discrepancy are the following: (1) the GFP signal has weaker tissue penetration and greater attenuation compared to the nanoprobe signal; (2) the nanoprobe may be released and subsequently label other cells *in vivo*.

### 3.3 Imaging immune cell rejection dynamics

**Figure 3:**
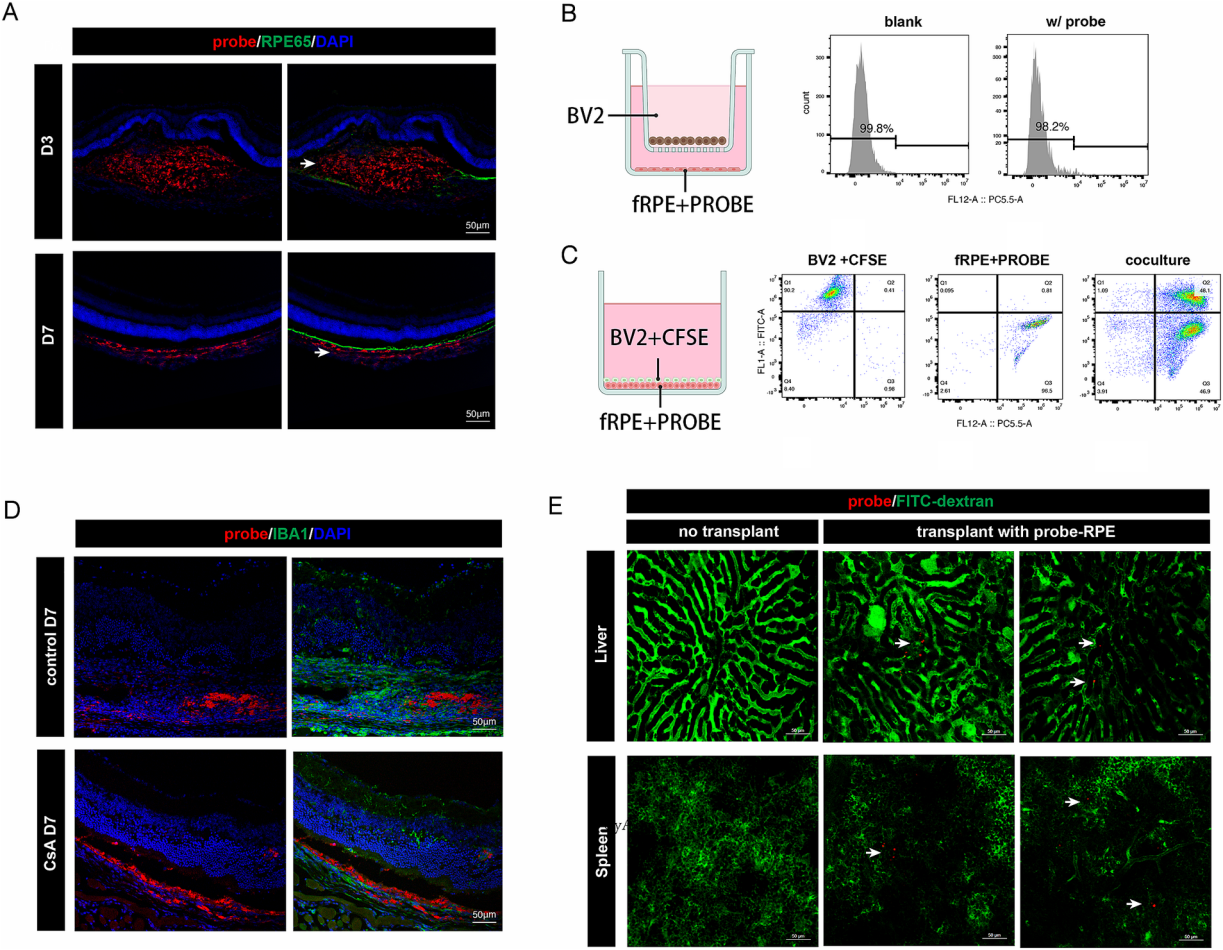
Distribution dynamics of probe signal *in vivo* and *in vitro*. **(A)** Immunofluorescence staining of RPE65 in retinal cryosections of mice injected subretinally with nanoprobe-labeled cells. The distribution of the nanoprobe signal (white arrow) transferred from the subretinal space to the choroid layer. Scale bar, 50 μm. **(B)** Experimental scheme for the non-contact co-culture assay. Flow cytometry analysis showing the proportion of probe-labeled BV2 microglial cells. **(C)** Experimental scheme for the contact co-culture assay. Flow cytometry analysis showing the proportion of CFSE/probe-labeled cells. **(D)** Immunofluorescence staining of IBA1 in retinal cryosections of mice injected subretinally with nanoprobe-labeled cells with or without intraperitoneal injection of cyclosporin A (CsA). Scale bar, 50 μm. **(E)** Nanoprobe signals (white arrow) in liver and spleen captured *in vivo* (*n* = 3).

We examined the distribution of the probe signal by fluorescence staining of the post-transplantation tissue sections. Our findings revealed that, on D3 post-transplantation, the probe signal was localized in the subretinal space of the mouse eye, whereas by the end of the first week, the probe signal had migrated to the choroid and co-stained with the myeloid marker IBA1 (Figure 3A). We hypothesized that this phenomenon might be attributed to the phagocytosis of cell-released probes or cellular debris by macrophages. To evaluate this hypothesis, we conducted a non-contact co-culture of nanoprobe-labeled and non-nanoprobe-labeled RPE cells with BV2 microglial cells for 48 h, followed by flow cytometry analysis of the BV2 cells. The analysis revealed the absence of nanoprobe-positive BV2 cells, suggesting that the nanoprobe remained stably associated with the RPE cells (Figure 3B). Moreover, in a contact co-culture experiment involving carboxyfluorescein diacetate succinimidyl ester (CFDA-SE)-labeled BV2 cells and nanoprobe-labeled RPE cells, we observed that the majority of CFSE-positive cells became nanoprobe/CFSE double-positive, indicating the successful transfer of the nanoprobe to the BV2 cells (Figure 3C). The aforementioned results demonstrated that the nanoprobe-labeled transplanted cells can release the nanoprobe, which is then transferred to adjacent cells by phagocytosis.

We performed nanoprobe-labeled RPE cell transplantation in immunosuppressive mice and found that the probe signal remained in the donor cells for up to 7 days post-transplantation (Figure 3D). This finding further confirms that the distribution of the nanoprobe signal is associated with the extent of graft rejection. Additionally, *in vivo* imaging of the liver and spleen, which are peripheral immune-related organs, revealed the presence of a small amount of nanoprobe signal in both organs 2-month post-transplantation (Figure 3E). This is the first evidence of systemic immune rejection following RPE transplantation. Together, the above findings suggest that long-term monitoring of nanoprobe-labeled cells can reveal dynamic changes within the transplant rejection microenvironment.

### 3.4 Single-cell dissection of the donor cell-host interaction landscape in RPE transplantation

**Figure 4:**
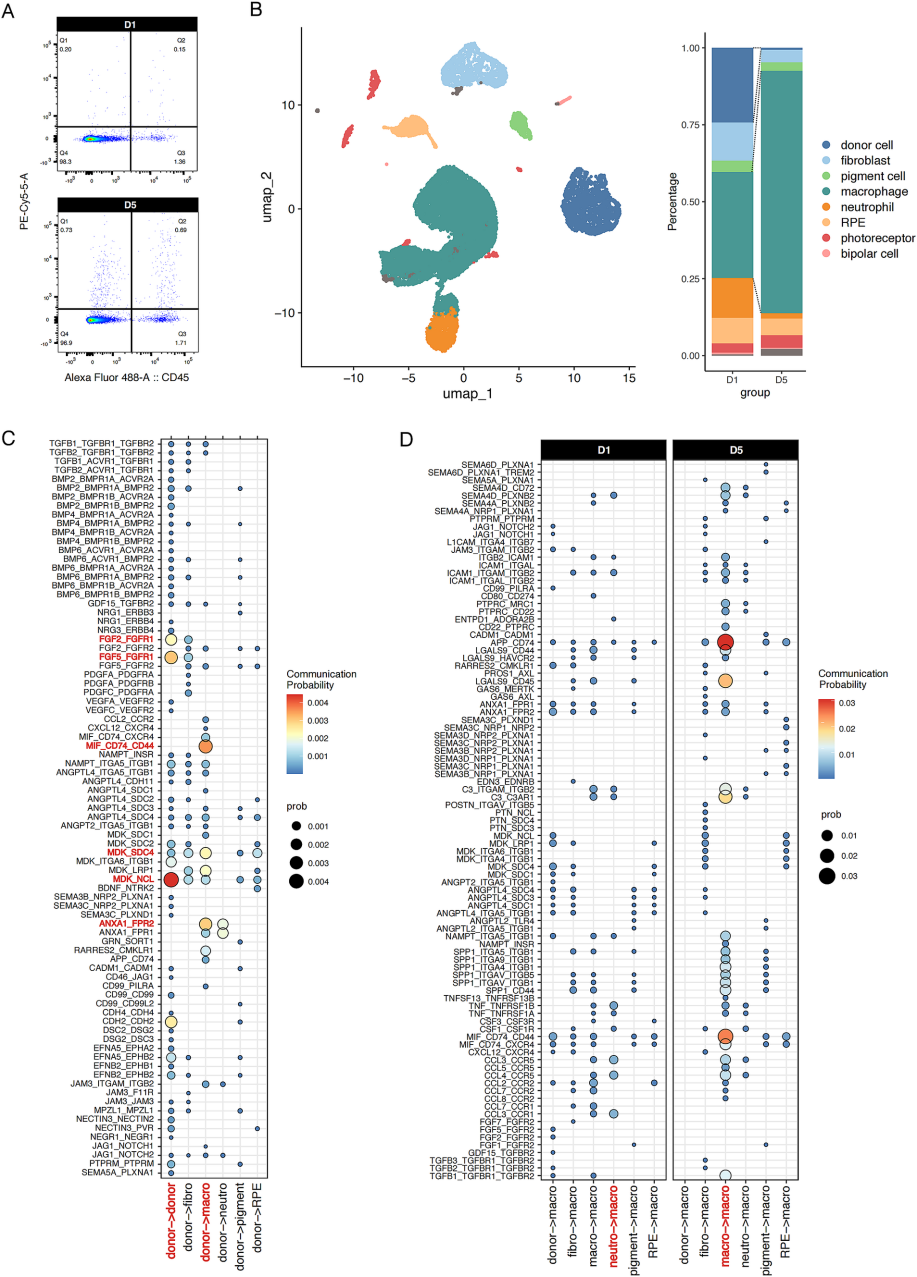
Single-cell intercellular communication between donor fRPE cells and transplantation niche cells. **(A)** Flow cytometry analysis of CD45^+^ and Probe^+^ cells in retina/RPE/choroid at D1 and D5 after transplantation. **(B)** Probe^+^ live cells were subjected to scRNA-seq analysis. UMAP plot of Probe^+^ cells from mice after transplantation showing 8 clusters. Stacked bar plot showing the composition of different cell types in Probe^+^ cells at D1 and D5. **(C)** Dot plot illustrating donor cell communicating with each cluster. **(D)** Dot plot illustrating macrophage activated by each cluster at D1 and D5. The communication probability of each group was represented by dot size and color intensity.

We performed scRNA-seq analysis on sorted nanoprobe-positive cells at various post-transplantation time points. Our analysis revealed that donor cells constituted 24% of the population on D1 post-transplantation. However, by D5, the proportion of probe-labeled donor cells had markedly decreased, being replaced by macrophages, which increased to 76% (Figure 4A). This finding further supports the involvement of phagocytosis in the primary mechanism of probe transfer between cells. Moreover, macrophage-mediated intrinsic immunity may play an important role in early graft rejection.

In addition, the scRNA-seq data provide valuable insights into the rejection microenvironment (Figure 4B). Thus, we performed single-cell intercellular communication analysis to investigate the initiators of immune rejection and the mechanisms underlying macrophage-mediated immune rejection. CellChat analysis revealed that the donor RPE cells strongly respond to autocrine midkine (MDK) and fibroblast growth factor (FGF) signaling. Concomitantly, donor cells significantly contribute to macrophage/neutrophil activation *via* annexin A1 (ANXA1) and MDK signaling (Figure 4C). ANXA1 and MDK are recognized as endogenous anti-inflammatory molecules that suppress immune activity and exacerbate tumor progression. The localized enrichment of immunosuppressive signals within grafts may seem contradictory to established paradigms, but they can act as signaling molecules for efferocytosis, mediating the phagocytosis of M2 macrophages and the formation of neutrophil extracellular traps. Thus, the above results highlight the double-sided nature of suppressive immune cells in the early transplantation microenvironment.

We found that macrophage inflammatory signals predominantly originate from neutrophils, as exemplified by cytokines such as CCL and TNF. However, following the attenuation of neutrophil-derived signals post-acute transplantation phase, these signals transition into macrophage autocrine signaling, thereby initiating a signaling cascade that promotes macrophage hyperactivation (Figure 4D). These findings further highlight the heterogeneous nature of transplantation-associated macrophages.

## 4 Discussion

**Figure 5:**
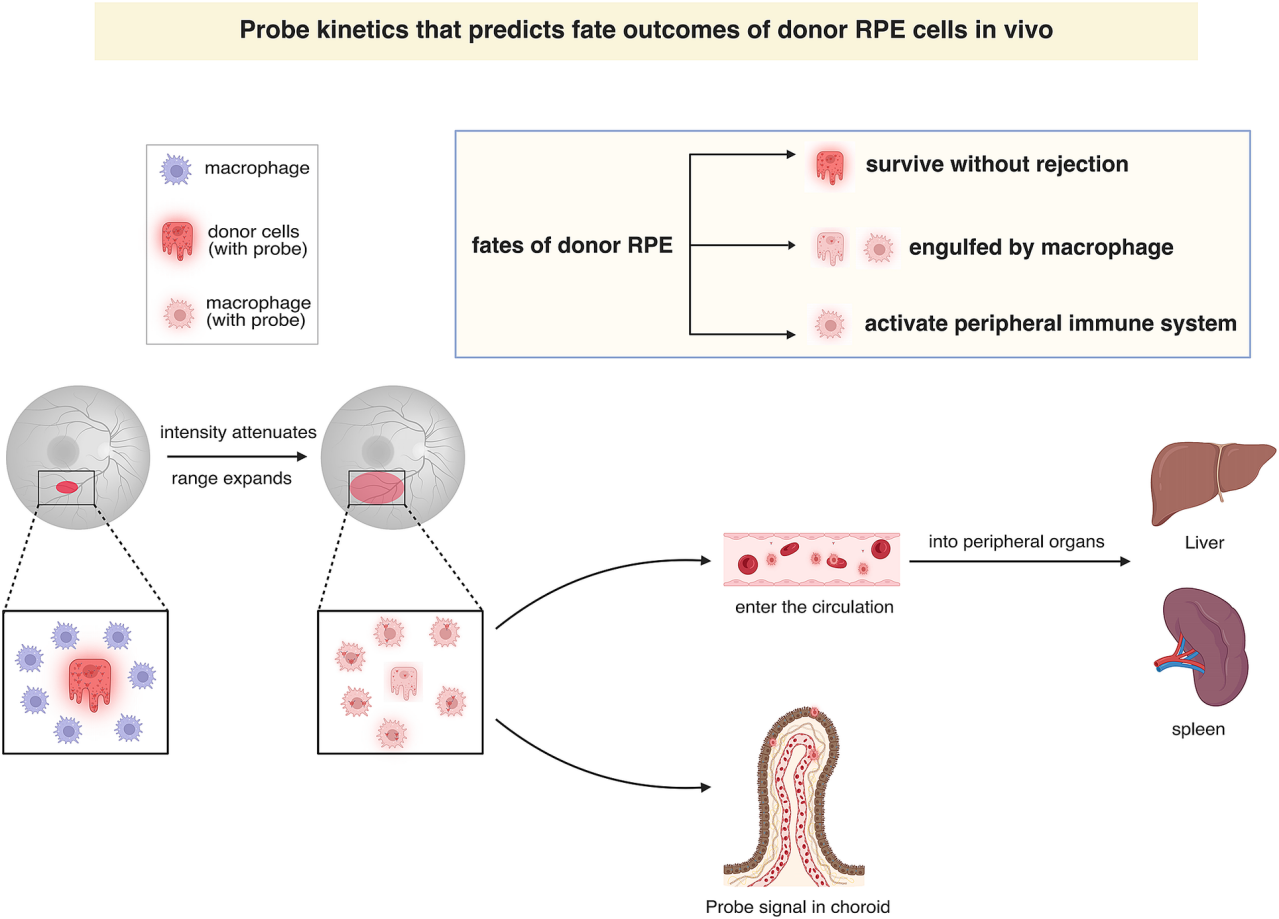
Graphical illustration of probe kinetics that predicts fate of donor RPE cells *in vivo*. In fundus fluorescence imaging, the attenuation and dispersion of the probe signal in the prolonged monitoring indicate that donor cells are engulfed by macrophages, which subsequently reside *in situ* or migrate to the spleen and liver through the bloodstream. The fate of donor cells can be categorized into three distinct groups according to the pathways followed: (1) cells functionally integrating into the host RPE layer without being rejected; (2) cell debris being phagocytosed by tissue-resident macrophages and residing within the retina or choroid; and (3) those activating the peripheral immune system by the circulating probe-labeled macrophage.

In this study, we have developed an imaging nanoprobe designed to monitor the fate of donor cells during RPE transplantation (Figure 5). Using NIR ratiometric imaging, fundus fluorescence imaging, and immunofluorescence staining techniques, we have demonstrated that the nanoprobe signal not only can be used to label the transplanted cells but can also be used to effectively track the turnover of donor cells (or cell debris) in real-time throughout the RPE transplantation process. As donor cells undergo phagocytosis or antigen presentation, the probe signal is relocated to phagocytes or antigen-presenting cells (APCs), thereby propagating the signal through the cellular hierarchy. Additionally, through long-term observation, we have monitored the nanoprobe signal in the living liver and spleen, demonstrating the biostability and optical sensitivity of this nanoprobe.

In RPE cell transplantation, monitoring residual donor RPE cells and immune rejection remains a significant challenge in assessing therapeutic efficacy. Previous clinical studies have used pigmentation in fundus photography and the reflective signal over the RPE in OCT as indicators of residual donor cells. However, there is increasing evidence showing that the pigmentation may be a temporal condition not accurately indicating the functional characteristics of the RPE (20–22). Preclinical studies have shown that overlying cell clumps may be associated with high risk of immune rejection. In terms of clinical monitoring for immune rejection, clinical studies of RPE transplantation typically employ strategies akin to those used in large organ transplantation, such as the analysis of circulating T cells (23–25). However, blood analysis appeared to lack sensitivity in the case of RPE transplantation. In this study, the range and intensity of the probe signal can be used to accurately assess the degree of immune rejection and the residual presence of donor cells, respectively.

In addition, we generated a single-cell atlas of RPE transplantation microenvironment by sorting probe-labeled cells. Our analysis identified an extensive array of cellular communication signaling molecules and potential APCs which acquire probe molecules through phagocytosis. This dataset serves as a reservoir of potential strategies for regulating RPE graft rejection and facilitating the functional integration of donor RPE cells.

Notably, in our single-cell analysis, T cell clusters were not detected, likely due to the lack of phagocytic ability of T cells. Our subsequent single-cell transcriptomic study of CD45 + cells showed that T cells accounted for only approximately 2% of these cells, suggesting that T cell-mediated adaptive immunity plays a limited role in RPE graft rejection (unpublished, Supplementary Figure S2). This finding also implies that the use of clinical-grade immunosuppressive agents, such as cyclosporine A, may have limited efficacy in the long-term management of RPE transplantation. Furthermore, our results indicate that macrophages not only function as APCs but also act as effector cells that directly reject grafts by recognizing “eat me” signals from donor cells and then instigating a deleterious cycle of immune hyperactivation.

In conclusion, a modified NIR fluorescent nanoprobe has been developed for the noninvasive monitoring of donor cells in RPE transplantation. The distribution and intensity of the nanoprobe signal, as determined by real-time imaging, may reflect varying fates of RPE grafts, providing a novel approach for the clinical evaluation of graft survival and immune rejection. Additionally, scRNA-seq analysis of nanoprobe-labeled cells improved our understanding of the RPE transplantation microenvironment and contributes to advance current prevention and therapeutic strategies for the chronic loss of transplanted RPE cells.

## Data Availability

The datasets presented in this study can be found in online repositories. The names of the repository/repositories and accession number(s) can be found at: https://ngdc.cncb.ac.cn/gsa, CRA019724.

## Supplementary material

Supplementary Figure S2 Results of GO enrichment analysis of differentially expressed genes (|log2FC| > 1 and *p* < 0.05).
