# Supplementary figures and images for: Near-infrared fluorescent nanoprobe enables noninvasive, longitudinal monitoring of graft outcome in RPE transplantation
Source: Front Med (Lausanne). 2025 May 9;12:1583790. doi: 10.3389/fmed.2025.1583790 (PMC12098337; doi:10.3389/fmed.2025.1583790)

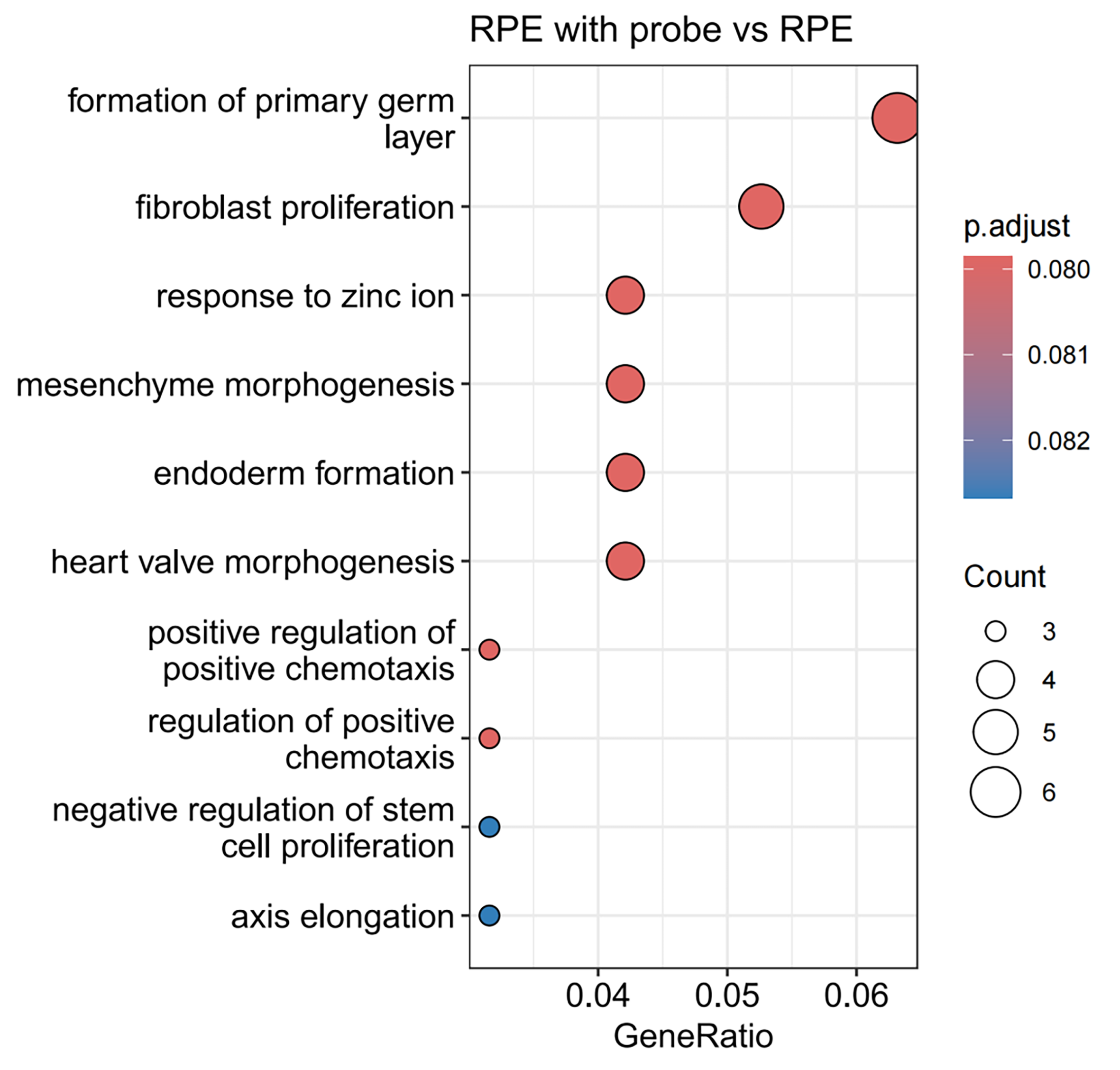

Supplement: Supplementary Figure S2 — Results of GO enrichment analysis of differentially expressed genes (|log2FC| > 1 and p < 0.05). [file Image_1.TIF]
