# Supplementary material for: Near-infrared fluorescent nanoprobe enables noninvasive, longitudinal monitoring of graft outcome in RPE transplantation
Source: Front Med (Lausanne). 2025 May 9;12:1583790. doi: 10.3389/fmed.2025.1583790 (PMC12098337; doi:10.3389/fmed.2025.1583790)
Supplement: Supplementary file 2 [file Data_Sheet_1.PDF]

Supplementary Figure S1

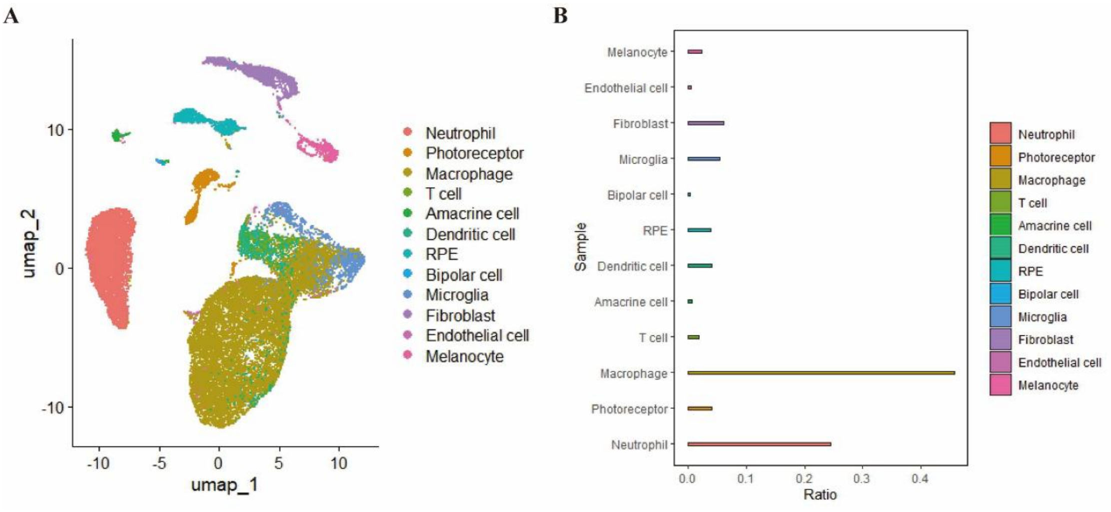

Fig S1. Cell composition in single-cell transcriptomic study of CD45$^+$ cells after retinal pigment epithelium cells transplantation. (A) CD45$^+$ live cells were subjected to scRNA-seq. UMAP plot of CD45$^+$ cells from mice after transplantation showing 12 clusters. **(B)** Stacked barplot showing the composition of different cell types in CD45$^+$ cells.

Supplementary Table S1.

No.	Gene name	log2FoldChange	padj
1.	ENSG00000284776	-8.36	0.000
2.	SLC16A8	1.58	0.000
3.	IGF2	-1.11	0.000
4.	NGFR	-2.08	0.000
5.	GNG2	-1.67	0.000
6.	LOC102724701	22.93	0.000
7.	SERF1B	1.08	0.001
8.	CDH13	-1.59	0.002
9.	MYH3	-1.19	0.006
10.	NPIPB11	1.23	0.008
11.	SLX1B	1.15	0.009
12.	DUSP2	-1.13	0.009
13.	ABCG1	1.28	0.009
14.	HEY1	-1.14	0.009
15.	PATL2	2.41	0.011
16.	TMEM200A	-1.11	0.011

17.	ENSG00000255126	1.97	0.011
18.	ZNF625-ZNF20	-1.80	0.011
19.	SNAI2	-1.30	0.015
20.	SCX	1.06	0.019
21.	PLAC9	1.27	0.020
22.	NPIPB3	1.01	0.023
23.	LINC03040	1.19	0.026
24.	ENSG00000258504	1.05	0.032
25.	TNFSF13	1.08	0.042
26.	C5orf46	1.21	0.044
27.	P4HA2-AS1	-2.60	0.047
28.	ENSG00000273784	-2.30	0.047
29.	ENSG00000250461	1.83	0.049
30.	ACTA1	-1.09	0.049
31.	ENSG00000279839	2.99	0.054
32.	ENSG00000227733	1.40	0.057
33.	MMP9	5.29	0.057
34.	ENSG00000277801	-2.53	0.063
35.	NPIPB12	1.07	0.065
36.	DNAJC14	-1.13	0.066
37.	ENSG00000263276	1.09	0.067
38.	SLC30A2	-1.40	0.071
39.	ENSG00000259627	1.43	0.071
40.	SULT1E1	3.61	0.071
41.	CSF1R	1.35	0.071
42.	ENSG00000258461	-4.12	0.091
43.	TRIM55	-1.26	0.091
44.	COMTD1	1.04	0.097
45.	WNT11	1.09	0.101
46.	NAMPTP1	-2.10	0.105
47.	UTS2R	1.56	0.110
48.	MESP1	1.36	0.110
49.	FLJ40194	-2.65	0.110
50.	NICOL1	1.07	0.120
51.	LIF-AS2	1.91	0.120
52.	ENSG00000277744	1.55	0.130
53.	FAM138F	5.13	0.130
54.	GOLGA8O	-4.26	0.140

55.	ENSG00000276698	2.01	0.142
56.	ENSG00000270696	7.34	0.142
57.	TBC1D3C	2.00	0.153
58.	MEF2B	1.50	0.160
59.	ENSG00000287650	1.57	0.170
60.	ENSG00000273711	1.52	0.170
61.	LRRTM2	1.18	0.170
62.	NPTX1	-1.07	0.172
63.	NUPR1	1.04	0.175
64.	SULT1A2	1.21	0.175
65.	KLK10	1.13	0.176
66.	ENSG00000232807	-1.24	0.180
67.	MIR302CHG	2.28	0.180
68.	MIR181A2HG	1.02	0.182
69.	ZMYND15	4.74	0.189
70.	SLFN11	1.93	0.189
71.	ENSG00000288550	-3.33	0.192
72.	H2BC26	-1.19	0.198
73.	ARSJ	-1.08	0.198
74.	ENSG00000255629	1.84	0.208
75.	ENSG00000288814	2.23	0.215
76.	ENSG00000224645	2.57	0.215
77.	TPM1-AS	1.17	0.215
78.	ENSG00000250899	-1.39	0.215
79.	ENSG00000288721	2.78	0.215
80.	BGLAP	1.13	0.216
81.	PKD1P5	-1.86	0.216
82.	MT1G	-2.34	0.216
83.	ENSG00000276791	1.99	0.216
84.	LOC124903317	-1.17	0.220
85.	C8orf34	1.37	0.220
86.	IL21-AS1	1.94	0.222
87.	LOC400499	1.44	0.223
88.	ATP6V0E2-AS1	1.26	0.223
89.	BEST4	1.23	0.226
90.	NAV2-AS1	-3.55	0.231
91.	LOC574538	1.19	0.231
92.	LRCOL1	1.68	0.231

93.	GPM6A-DT	2.64	0.233
94.	FANCB	-1.16	0.233
95.	ENSG00000291272	-1.74	0.233
96.	FDCSP	1.49	0.236
97.	RSPO3	1.97	0.240
98.	ENSG00000248101	1.10	0.240
99.	ENSG00000267059	-7.45	0.242
100.	MYH7	-1.04	0.242
101.	ENSG00000285851	-1.89	0.246
102.	ENSG00000280649	1.13	0.249
103.	ENSG00000287286	-1.11	0.250
104.	ENSG00000284602	2.25	0.254
105.	ENSG00000283149	-1.38	0.254
106.	TIE1	-1.32	0.254
107.	RAG1	-1.61	0.254
108.	MORF4L1P1	-1.09	0.254
109.	NPFF	1.11	0.254
110.	UPK1B	-1.95	0.254
111.	TSTD2	-2.10	0.254
112.	ENSG00000290948	-1.30	0.256
113.	FBXL16	-1.04	0.259
114.	TVP23C-CDRT4	-2.07	0.259
115.	SYNJ2BP-COX16	1.27	0.260
116.	PCAT1	1.03	0.260
117.	ENSG00000232386	1.65	0.260
118.	ZC3H12D	-1.82	0.265
119.	AMPH	-1.38	0.266
120.	ESR1	2.81	0.270
121.	OAS1	1.14	0.274
122.	ZNF567	-1.01	0.279
123.	MYH11	3.27	0.279
124.	PCARE	1.48	0.287
125.	C4A-AS1	-7.03	0.289
126.	LINC00313	1.50	0.289
127.	ACKR3	-1.02	0.289
128.	ENSG00000273218	2.39	0.289
129.	LOC118567325	1.23	0.290
130.	ENSG00000274370	1.77	0.296

131.	ENSG00000285184	-1.10	0.299
132.	ENSG00000224282	1.90	0.300
133.	LINC00685	1.12	0.300
134.	ENSG00000288632	-1.56	0.300
135.	ENSG00000260879	-2.59	0.300
136.	PLGLB2	1.27	0.306
137.	LOC102724594	10.66	0.306
138.	H1-10-AS1	1.05	0.308
139.	LRTOMT	1.05	0.309
140.	ENSG00000277959	1.82	0.309
141.	SFTPD	2.03	0.311
142.	ABCA17P	1.71	0.313
143.	ENSG00000260816	1.14	0.313
144.	ENSG00000286623	1.84	0.318
145.	SLITRK1	1.71	0.318
146.	RAMP1	1.29	0.318
147.	ENSG00000271384	2.25	0.322
148.	ENSG00000283294	1.74	0.324
149.	ENSG00000205236	-2.00	0.324
150.	KCNQ1	1.21	0.324
151.	OTUD7A	2.11	0.334
152.	NRCAM	-1.85	0.336
153.	NEFM	1.68	0.336
154.	OR7E14P	1.83	0.336
155.	ENSG00000238260	1.61	0.338
156.	LOC441239	1.37	0.341
157.	CCND2-AS1	1.00	0.343
158.	ENSG00000272954	2.04	0.343
159.	TMX2P1	-1.79	0.344
160.	ENSG00000273355	-1.56	0.345
161.	ENSG00000260007	-1.81	0.346
162.	TEX41	1.45	0.346
163.	OASL	2.33	0.346
164.	LINC01736	1.66	0.346
165.	CTBP1-AS	-1.24	0.346
166.	C1QTNF5	1.12	0.346
167.	TEDC1	1.06	0.346
168.	SHBG	1.39	0.355

169.	ENSG00000279278	-1.71	0.355
170.	CCN5	1.85	0.355
171.	C16orf95-DT	1.48	0.355
172.	MT1X	-1.27	0.357
173.	GUCY1B1	-1.18	0.358
174.	CASP10	1.36	0.358
175.	LOC102724684	-1.67	0.358
176.	AVIL	-1.03	0.358
177.	ENSG00000266378	1.02	0.358
178.	REEP1	1.05	0.362
179.	THBS1-IT1	-1.63	0.362
180.	LINC01943	-1.37	0.364
181.	ENSG00000287387	1.26	0.367
182.	ENSG00000270068	1.40	0.367
183.	ENSG00000258768	-1.24	0.367
184.	LINC01111	-1.88	0.367
185.	PCDHGB7	-1.42	0.370
186.	PTPN23-DT	1.12	0.371
187.	ENSG00000273321	1.34	0.371
188.	ENSG00000291201	-1.03	0.373
189.	SUGT1P4-STRA6LP	1.66	0.374
190.	IL16	-1.35	0.374
191.	ITGB2	2.89	0.374
192.	ENSG00000284946	-1.62	0.376
193.	LEKR1	1.68	0.380
194.	INA	-1.10	0.385
195.	GRB14	-1.43	0.391
196.	ENSG00000266456	1.67	0.391
197.	MARCHF4	-1.14	0.391
198.	MIR12136	1.69	0.391
199.	ENSG00000274444	1.94	0.396
200.	LCN10	1.52	0.397
201.	ENSG00000280219	1.18	0.398
202.	FGF10	2.54	0.399
203.	ENSG00000240207	1.04	0.399
204.	LAPTM5	1.52	0.400
205.	OTULIN-DT	1.86	0.400
206.	ENSG00000261512	-1.16	0.400

207.	LINC00930	-1.53	0.401
208.	CASQ2	-1.01	0.401
209.	ZBTB47-AS1	1.07	0.406
210.	KLHDC7B	1.31	0.408
211.	ENSG00000258559	-1.70	0.408
212.	HHIPL2	-1.22	0.413
213.	KRT13	-1.26	0.413
214.	HRK	-1.03	0.413
215.	SCHIP1	-1.16	0.413
216.	ENSG00000224066	1.61	0.413
217.	MOV10L1	1.22	0.413
218.	ZNF492	-1.21	0.414
219.	NBPF25P	-1.28	0.414
220.	ENSG00000260495	1.20	0.414
221.	ENSG00000286078	3.25	0.414
222.	ENSG00000285702	1.17	0.414
223.	SLC22A4	-1.43	0.416
224.	SCT	1.02	0.417
225.	MAP3K4-AS1	-1.06	0.418
226.	ENSG00000276533	1.14	0.418
227.	ENSG00000276250	1.52	0.418
228.	RNF17	1.18	0.420
229.	DRICH1	1.57	0.421
230.	ENSG00000275185	1.35	0.421
231.	PTPRT	3.25	0.422
232.	ENSG00000254829	1.50	0.426
233.	ENSG00000279103	1.11	0.426

Table S1. All differentially expressed genes between fetal retinal pigment epithelium cells and probe-labeled fetal retinal pigment epithelium cells (-2< FoldChange <2).
